# The Potential of *Chaetoceros muelleri* in Bioremediation of Antibiotics: Performance and Optimization

**DOI:** 10.3390/ijerph18030977

**Published:** 2021-01-22

**Authors:** Amin Mojiri, Maedeh Baharlooeian, Mohammad Ali Zahed

**Affiliations:** 1 Department of Civil and Environmental Engineering, Graduate School of Advanced Science and Engineering, Hiroshima University, Higashihiroshima 739-8527, Japan; 2 Department of Marine Biology, Faculty of Marine Science and Oceanography, Khorramshahr University of Marine Science and Technology, Khorramshahr 64199-34619, Iran; bbenicka@yahoo.com; 3 Faculty of Biological Sciences, Kharazmi University, Tehran 15719-14911, Iran; zahed51@yahoo.com

**Keywords:** antibiotics, bioremediation, microalgae, ofloxacin, sulfamethoxazole, toxicity

## Abstract

Antibiotics are frequently applied to treat bacterial infections in humans and animals. However, most consumed antibiotics are excreted into wastewater as metabolites or in their original form. Therefore, removal of antibiotics from aquatic environments is of high research interest. In this study, we investigated the removal of sulfamethoxazole (SMX) and ofloxacin (OFX) with *Chaetoceros muelleri*, a marine diatom. The optimization process was conducted using response surface methodology (RSM) with two independent parameters, i.e., the initial concentration of antibiotics and contact time. The optimum removal of SMX and OFX were 39.8% (0.19 mg L^−1^) and 42.5% (0.21 mg L^−1^) at the initial concentration (0.5 mg L^−1^) and contact time (6.3 days). Apart from that, the toxicity effect of antibiotics on the diatom was monitored in different SMX and OFX concentrations (0 to 50 mg L^−1^). The protein (mg L^−1^) and carotenoid (μg L^−1^) content increased when the antibiotic concentration increased up to 20 mg L^−1^, while cell viability was not significantly affected up to 20 mg L^−1^ of antibiotic concentration. Protein content, carotenoid, and cell viability decreased during high antibiotic concentrations (more than 20 to 30 mg L^−1^). This study revealed that the use of *Chaetoceros muelleri* is an appealing solution to remove certain antibiotics from wastewater.

## 1. Introduction

Efficient wastewater treatment is vital for both industries and municipalities. There are several kinds of pollutants in wastewater, including pharmaceuticals [1]. Several studies have shown that the accumulation of pharmaceutical ingredients in water bodies may have a negative effect on the health of humans and other organisms [2]. Moreover, because of their lipophilic nature, some of these ingredients may be adsorbed sediments and particles, and bioaccumulate along the trophic chain. Antibiotics is one class of broadly consumed pharmaceuticals [3]. Antibiotics have been widely applied in human disease treatment, livestock, and poultry farms. Antibiotic usage has increased from 21.1 billion defined daily doses to 34.8 billion defined daily doses, increasing around 65% between 2000 and 2015 [4]. Moreover, the total antibiotic consumption for livestock was 63,151 tons in 2015, which will increase by 15% in 2030 [5]. Almost 30% to 90% of antibiotics applied by an organism is excreted [6].

Increasing antibiotic consumption results in rising environmental pollution [7] since antibiotics can affect organisms’ microbiome, as well as the microbiological balance in ecosystems. Additionally, the presence of antibiotics in the environment causes antibiotic resistance genes to grow, which drags human health into a dangerous condition [8]. Sulfamethoxazole (SMX) is among the most frequently detected antibiotics in aquatic environments due to its wide application in human and veterinary medicine [9,10]. Ofloxacin (OFX) is the third generation of quinolones antibiotics, which is widely applied to treat human and animal diseases. OFX has been extensively detected in water bodies around the world, ranging from 0.5 ng/L to 30 mg/L [11]. These antibiotics are among the high resilience pharmaceutical micropollutants in the environment that affect aquatic life [12]. OFX, as a fluoroquinolone, are subjected to biotransformation by some microorganisms (such as microalgae and fungi) during reactions comprised of hydroxylation, defluorination, and decarboxylation of piperazine ring partial removal [13].

Therefore, eliminating these antibiotics with conventional wastewater treatment processes are limited. Furthermore, removing antibiotics generally involves high cost [14]. Thus, finding effective treatment methods has attracted research interest [10]. One of the most effective methods is an algae-based system.

Using microalgae as an environmentally friendly and low-cost water treatment technique has been considered as a way to eliminate pollutants from water bodies [15]. Amenorfenyo et al. [15] accounted several advantages for microalgae-based treatment techniques, including the removal of nutrients and pollutants from wastewater, releasing oxygen (which may be consumed by bacteria in the wastewater), and fixing CO_2_. Furthermore, microalgae are considered to have high growth rates, high photosynthetic effectiveness, wide adaptability, and good potential to eliminate contaminants from wastewater [16]. One of the abundant types of phytoplankton in the ocean is the marine diatom. Marine diatoms are considered for almost 40% of the global oceanic organic carbon production per year and play a role for up to 25% of the global CO_2_ fixation [17]. *Chaetoceros muelleri* (Table 1) has been commonly applied as live feed in fish aquaculture due to its good nutritional properties [18]. Only a few research studies have reported to use marine diatoms for wastewater treatment [13] and especially antibiotic removal. Therefore, this study was conducted to fill this gap. The goals of the study were as follows: (1) to remove antibiotics using a photobioreactor (contained *Chaetoceros muelleri*); (2) to optimize the surface methodology (RSM) response; and to measure (3) toxicity effects of antibiotic concentrations on *Chaetoceros muelleri*.

## 2. Materials and Methods

**Figure 1:**
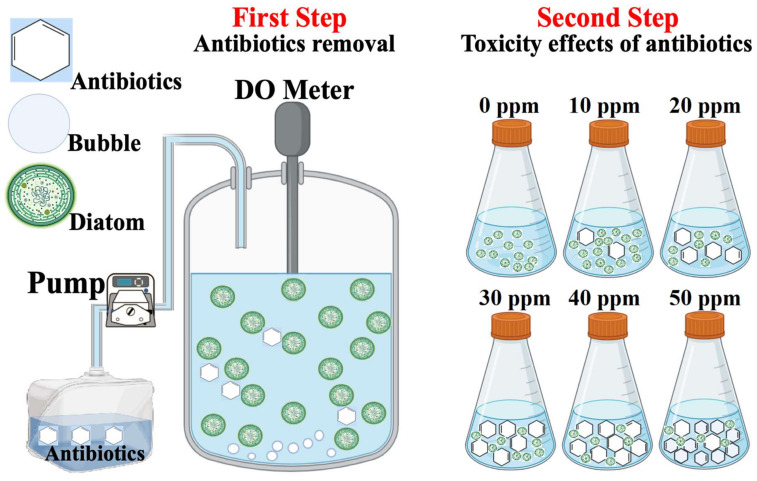
Schematics of the study.

Based on the study by Mojiri et al. [18], synthetic wastewater was contaminated with SMX and OFX. SMX and OFX (Table 2) were purchased from Sigma-Aldrich Co. (Petaling Jaya, Malaysia) with a purity of ≥98%. To prepare stock solutions of 1 g L^−1^, each antibiotic was dissolved in distilled water. Such stock solutions were then diluted to obtain antibiotic concentrations. *Chaetoceros muelleri* was obtained from the photobioreactor in our laboratory, which operated at room temperature (25 °C) with light:dark cycles of 12:12 h (light was set around 60 to 70 μmol photons m^−2^ s^−1^) [25] under an aeration rate of 0.4 L/min [2]. This study had two stages: the removal of antibiotics and ecotoxicology effects of antibiotics on algae (Figure 1).

### 2.1. Experimental Setup

Under a constant light of 66 μmol photons m^−2^ s^−1^ in the F/2 medium and artificial seawater, *Chaetoceros muelleri* was cultivated. The F/2 medium was included several compounds such as NaNO_3_, NaH_2_PO_4_, Na_2_H_2_EDTA, FeCl_3_·6H_2_O, MnCl_2_·4H_2_O, ZnSO_4_, Na_2_SiO_3_ etc., as described by [28]. Afterward, a certain amount (20 × 10^6^ cell mL^−1^) of marine diatom [29] was transferred to a 6 L (a lab-scale) bubble column photobioreactor at room temperature under white fluorescent light illumination (66 μmol photonsm^−2^ s^−1^) (González-González et al. [30]). The photobioreactor was a tank (width (7 cm), length (20 cm), and height (44 cm)) that had a working value of 6 L. The aeration was done from the bottom. Hydraulic retention time (day) and aeration rates were set at 2.8 and 0.4 based on our preliminary experiments, which were in line with Jiménez-Bambague et al. [31] and Sun et al. [32], respectively. Synthetic aqueous solution was produced by dissolving both antibiotics and artificial seawater.

### 2.2. Analytical Methods

High performance liquid chromatography (LC-20AT, Shimadzu International Trading Co., Ltd., Tokyo, Japan) was furnished with a UV detector. The detection wavelength was set at and 288 nm to monitor the concentrations of antibiotics, as described by Oh et al. [33] and Guo et al. [11]. The mobile phase was methanol and deionized water (1:1) with a flow rate of 1.0 mL min^−1^. The 3σ/s was [18] considered to assess the limit of detection (LOD).

### 2.3. Optimization Process

The removal efficacy of antibiotics was evaluated based on Equation (1). Two factors, including antibiotic concentrations and contact time, were considered as independent parameters.

(1) $$\mathrm{Removal}\;(\%)\;=\;\frac{\left(C_{i}-C_{f}\right)}{C_{i}}\;\times \;100,$$

where, *C_i_* and *C_f_* indicate the initial concentrations of antibiotics, and concentration of antibiotics in effluent after treatment, respectively.

The total concentrations of antibiotics ranged from 0.5 mg L^−1^ to 3 mg L^−1^ [32]. Contact time (day) varied 0.5 to 6.5 [34]. Design expert software (version 10.0) was employed to assess the response surface methodology (RSM) and central composite design (CCD) to optimize and to analyze the photobioreactor’s performance in eliminating antibiotics. The details of inputs and runs are presented in Table 1. Each factor contained three levels; hence, a quadratic model is a proper model (Equation (2)).

(2) $$Y=\;\beta_{0}+\;\sum_{j=1}^{k}\beta_{j}X_{j}+\;\sum_{j=1}^{k}\beta_{jj}X_{j}^{2}+\;\sum_{j}\sum_{<i=2}^{k}\beta_{ji}X_{i}X_{j}\;+e,$$

where *Y* defines antibiotic removal, the constant coefficient is presented by *β_0_*, *k* displays the number of factors, and *X_j_* and *X_i_* demonstrate the variables. Moreover, the interaction coefficients of the linear, quadratic, and second-order terms are presented by *β_j_, β_jj_,* and *β_ij_*, respectively.

### 2.4. Toxicity Effects of Antibiotics on Microalgae

Assessing the ecotoxicological effects of antibiotics at different antibiotic concentrations (0 to 50 mg L^−1^) were conducted in batch experiments from 0 to 8 days, as described by Chen et al. [35]. The experiments were conducted in 250 mL Erlenmeyer flasks comprised of 150 mL F/2 medium inoculated with microalgal cell (80 mg L^−1^) suspension under constant light of 66 μmol photons m^−2^ s^−1^. Total protein (mg L^−1^), carotenoid concentration (μg L^−1^), and cytotoxicity (%) were monitored in the triplicate as follows. Protein content was analyzed with the UV-Vis spectrophotometer at 595 nm, as described by Chia et al. [36] based on the Bradford method.

Total carotenoid concentration was monitored by a spectrophotometer (UV-1601PC, Shimadzu, Tokyo, Japan) at 470 nm, as described by Costache et al. [37] and based on the Lichtenthaler and Wellburn [38] equations (Equations (3 to 5)). The pigment was extracted with acetone after centrifugation (15 min and 6000 rpm) and kept in a cool and dark place for one day.

C*a* (μg L^−1^) = 11.47A_664_ − 0.4A_630,_ (3)

C*b* (μg L^−1^) = 24.36A_630_ − 3.73A_664,_ (4)

C_X + c_ = (1000A_470_ − 2.27C_a_ − 81.4C_b_)/227, (5)

where *C_X+c_* displays the total carotenoid concentration. The absorbance values (A) at a wavelength of 470, 630, and 664 nm are defined by *A_470_*, *A_630_,* and *A_664_*, where chlorophyll *a* and *b* are defined by *C_a_* and *C_b_*, respectively.

Cytotoxicity (%) was assessed as described by Namasivayam et al. [39]. The optical density (OD) was monitored using a spectrophotometer at 630 nm.

(6) $$\mathrm{Cytotoxicity}\;(\%)\;=\;\frac{\left(\mathrm{OD}\;\mathrm{of}\;\mathrm{individual}\;\mathrm{test}\;\mathrm{group}\right)100}{\left(\mathrm{OD}\;\mathrm{of}\;\mathrm{control}\;\mathrm{group}\right)}.$$

## 3. Results and Discussion

**Figure 2:**
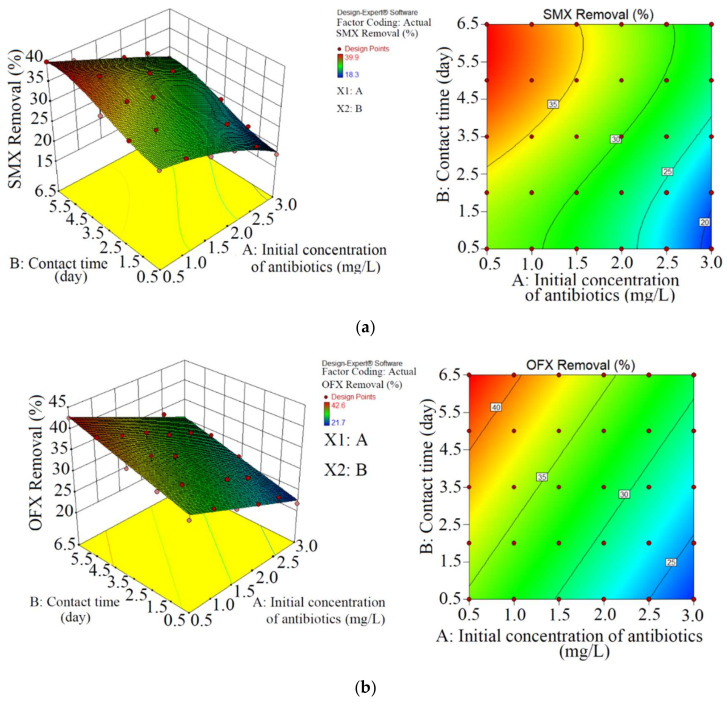
The 3D plots for the removal of SMX (**a**) and OFX (**b**) with diatom.

**Figure 3:**
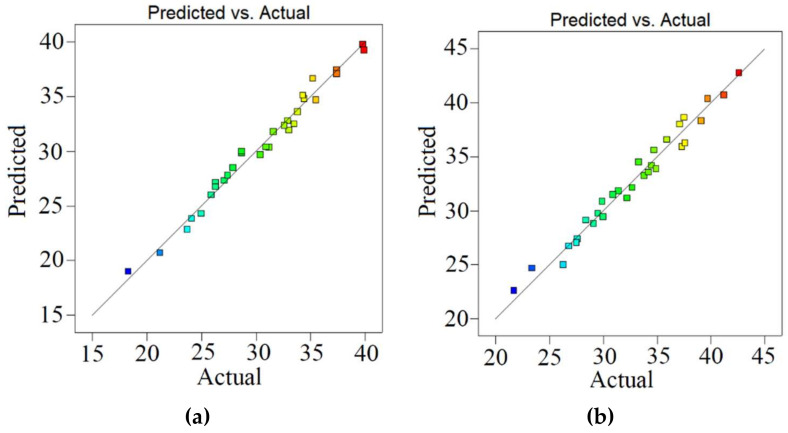
Model prediction versus experimental values for the removal of SMX (**a**) and OFX (**b**) with diatom.

Two steps were considered during the current study. In the first step, the SMX and OFX were removed by *Chaetoceros muelleri*. The elimination effectiveness was optimized by RSM, as shown in Table 3, Table 4, Figure 2 and Figure 3. During the second step, the toxicological effects of antibiotics were monitored. Thus, this comprehensive study about antibiotic removal using a marine diatom has not been reported in previous studies.

### 3.1. Removal of Antibiotics

As shown in Table 3 and Figure 2, the maximum abatement of SMX (39.9% or 0.20 mg L^−1^) was reached at contact time (5.0 days) and initial concentration of antibiotics (0.5 mg L^−1^). In addition, the minimum abatement of SMX (18.3% or 0.54 mg L^−1^) was reached at a contact time (0.5 days) and initial concentration of antibiotics (3.0 mg L^−1^). Bai and Acharya [40] reported that sulfamethoxazole was removed less than 60% after 14 days by a modified algae-mediated photolysis.

Apart from that, the maximum reduction of OFX (42.6% or 0.21 mg L^−1^) was obtained at a contact time (6.5 days) and initial antibiotic concentration (0.5 mg L^−1^). Moreover, the minimum OFX reduction (21.7% or 0.65 mg L^−1^) was obtained at a contact time (0.5 days) and initial antibiotic concentration (3.0 mg L^−1^). Further, Ref. [41] reported that while ofloxacin compounds considerably decreased through the biological treatment, it was still found in effluent in notable concentrations (0.64 μg L^−1^).

Antibiotic removal increased with increasing time from 0.5 days to 6.5–7 days during the research. Moreover, Maryjoseph and Ketheesan [42] expressed that contact time influenced the time available for soluble contaminant degradation. Gentili and Fick [43] reported one week for reaching optimum pharmaceutical with algal. In the study, OFX removal was slightly better than SMX removal. Bai and Acharya [40] expressed that SMX had a very persistent antibiotic that cannot efficiently be removed by algae. Almost 0.2 mg L^−1^ of SMX in 12 days was removed by *Scenedesmus obliquus* [44]. Yang et al. [45] removed 9.9 to 39.2% of OFX with *Scenedesmus obliquus*.

Using microalgae to remove micropollutants (i.e., antibiotics) is an environmentally friendly method that does not produce secondary pollutants [42]. Hena et al. [46] expressed that microalgae can uptake and accumulate pollutants within the cell for growth processes. Furthermore, microalgae use light energy, and organic and inorganic nutrients to develop and synthesize biocompounds that have a high aggregated nutritional value and therapeutic functions [47]. On the other hand, algae can convert pollutants to biomass and lipids for several useful applications [48].

Based on RSM optimization, optimum abatement of SMX and OFX were 39.8% (0.19 mg L^−1^) and 42.5% (0.21 mg L^−1^) at the initial concentration (0.5 mg L^−1^) and initial contact time (6.3 days). Figure 3 shows the plots of the experimental data compared to the predicted data, specifying a rational distribution of points around the X = Y line in a narrow area. Apart from Figure 3, the high value of *R^2^* (Table 4) displayed that RMS optimized the antibiotic removal in a logical way, which can be confirmed by Dolatabadi and Ahmadzadeh [49].

### 3.2. Toxicity of Antibiotics

**Figure 4:**
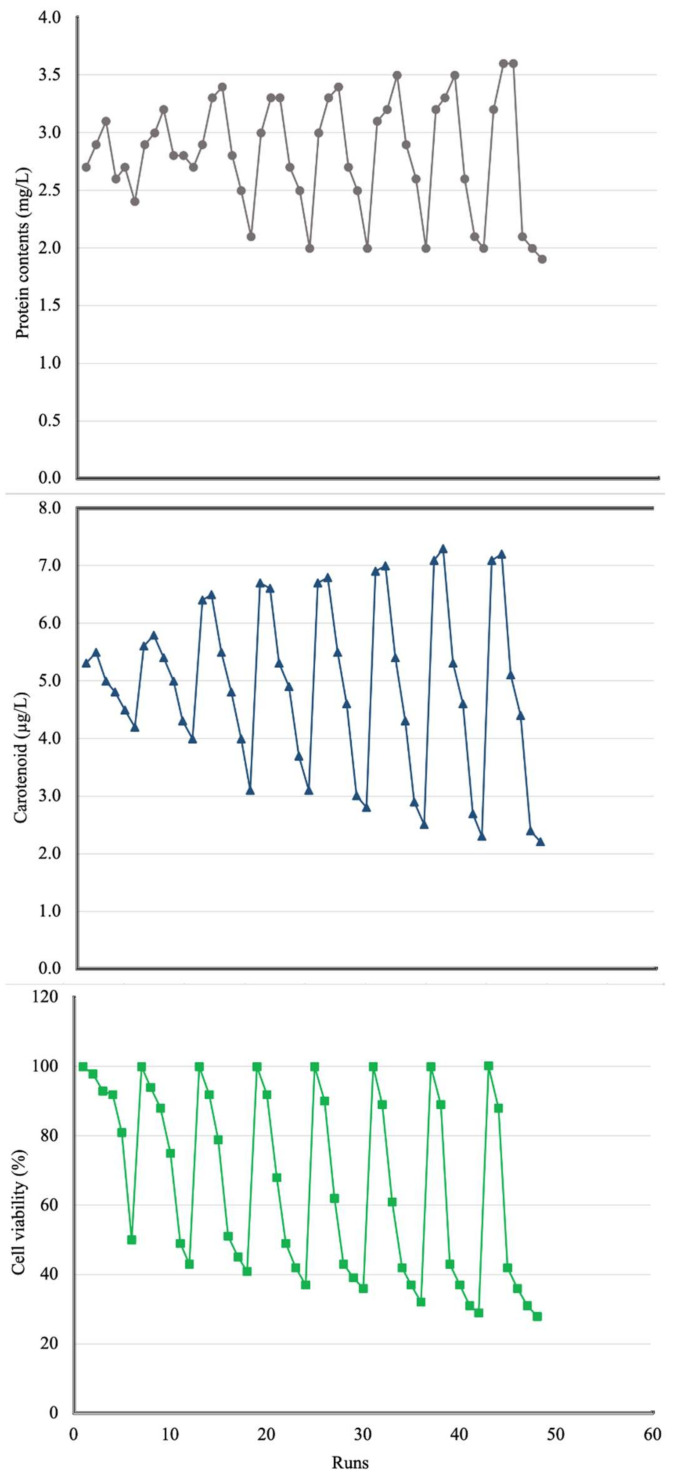
Toxicological effects of antibiotics on marine diatoms.

Some PPCPs can have a toxic effect on microalgae species during the removal of pharmaceuticals and personal care products (PPCPs) with algae-based systems [42]. In this case, the performance of microalgae in removing PPCPs may be reduced. For instance, Sendra et al. [50] stated that antibiotics (such as erythromycin) may have negative effects on freshwater and marine microalgae. Thus, the toxicity effects of these antibiotics at different concentrations on *Chaetoceros muelleri* were investigated. Table 5 and Figure 4 display details about antibiotic toxicity effects on marine diatoms.

Changes in protein content and carotenoid concentration are important indicators to monitor the toxicity effects of pharmaceuticals micropollutants on algae [51,52].

Details about toxicity experiments are summarized in Table 3. Runs 1, 7, 13, 19, 25, 31, 37, and 43 were blank and antibiotics were not added to the reactor for toxicity analysis. In runs 2, 8, 14, 20, 26, 32, 38, and 44, 10 mg L^−1^ of antibiotics were added and samples were taken after 1, 2, 3, 4, 5, 6, 7, and 8 days, respectively. Figure 4 shows the protein content, cell viability, and carotenoid concentrations in each run of Table 3.

As shown in Figure 4, the maximum protein content (3.6 mg L^−1^) was recorded at runs 44 and 45 (8 days and initial concentrations of antibiotics were 10 and 20 mg L^−1^). Moreover, the minimum protein content was 1.9 mg L^−1^ at run 48 (8 days and initial concentrations of antibiotics were 50 mg L^−1^). There was no huge difference between the maximum and minimum recorded protein contents. Liu et al. [53] stated that the total protein content (in marine diatom *Navicula incerta*) decreased from 17.7% to 46.7% when pharmaceutical concentrations increased. Apart from that, Yang et al. [45] expressed that xenobiotic-induced stress caused protein metabolism to defend against the stress affected by abiotic parameters. The results specified that the protein remained moderately stable. Increasing the protein content of diatoms at low antibiotic concentrations was justified by Chen et al. [54]. This increase was related to the increase in enzyme synthesis or other energy-producing fractions.

The maximum total carotenoid concentration (7.3 μg L^−1^) was reached at run 38 (7 days and initial concentrations of antibiotics were 10 mg L^−1^). This means that the total carotenoid concentration increased when the amount of antibiotic increased to 10 mg L^−1^. Moreover, the minimum total carotenoid concentration (2.2 μg L^−1^) was reached at run 48 (8 days and initial concentrations of antibiotics were 50 mg L^−1^). Then, the total carotenoid decreased when antibiotic concentration increased 10 mg L^−1^ to 50 mg L^−1^. Zhang et al. [51] expressed that low concentrations of pharmaceuticals had a positive role on chlorophyll *a*. Wang et al. [55] stated that the chlorophyll *a* in *Microcystis* increased at low pharmaceutical concentrations but was reduced at high pharmaceutical concentrations. Guo et al. [56] reported that high antibiotic concentrations could decrease the total chlorophyll and carotenoid contents of algal species from 2.4 units (109 mg L^−1^ cell^−1^) in control to 1.67 units after four days. Singh et al. [57] expressed that the antibiotic-induced photosystem stress might justify the decrease in total chlorophyll and carotenoid contents after antibiotic concentrations increased. Nie et al. [58] expressed that chlorophyll biosynthesis transformed after its exposure to high amounts of pharmaceuticals, leading to the inhibition growth of algae. In high antibiotic concentrations, the decrease in chlorophyll and carotenoid contents was justified with the reactive oxygen species (ROS)-mediated damage to the photosystem and chlorophyll biosynthesis in microalgae. Chlorophyll of cells may be used as a protective method to reduce the accumulated ROS in chloroplasts [54].

Increasing the concentration (mg L^−1^) of antibiotics from 10 to 20 did not have any significant effects on cell viability (cytotoxicity). Xiong et al. [59] stated that more than 10 mg L^−1^ of ofloxacin compounds could significantly inhibit microalgae growth and activity. Viability of *C. reinhardtii* decreased around 68% after 72 h of exposure to antibiotics, compared with the control (blank) [60]. Apart from that, the cell viability of microalgae decreased when diatoms were exposed to high antibiotic concentrations for more than four days. Mojiri et al. [2] stated that pharmaceuticals should penetrate the cell for its act, thus taking time to receive maximum PPCP impact on the cell.

## 4. Conclusions

Conventional treatment techniques for domestic sewage treatment plants do not efficiently eliminate antibiotics. Thus, we investigated the potential of *Chaetoceros muelleri* in eliminating sulfamethoxazole (SMX) and ofloxacin (OFX). The key achievements of the study are as follows:

1. Based on the RSM at the initial concentration (0.5 mg L^−1^) and contact time (6.3 days), the optimum removal of SMX and OFX were 39.8% (0.19 mg L^−1^) and 42.5% (0.21 mg L^−1^).
2. The RSM optimized the removal of antibiotics with diatoms in a logical way because of the high *R*^2^ value and rational distribution of experimental data compared to the predicted data.
3. Based on the toxicological effects of antibiotics on microalgae, total carotenoid concentration, protein and cell viability decreased at high antibiotic concentrations.

## Figures and Tables

**Table 1:** Advantages and uses of *Chaetoceros muelleri*.

Application	References	Application	References
Wastewater treatment	[2]	Providing high-value products (linoleic acid, and carbohydrates)	[19]
Application in food industry	[19]	High lipid content	[20]
High biomass for bioenergy	[21]	Aquaculture feed	[22]
Degradation of diethyl phthalate	[23]	Agricultural fertilizer	[24]

**Table 2:** Characteristics of antibiotics used in this study.

Compounds	CAS Number	Molecular Formula	Molecular Weight	References
SMX	732-46-6	C_10_H_11_N_3_O_3_S	253.28	[26]
OFX	82419-36-1	C_18_H_20_FN_3_O_4_	361.40	[27]

**Table 3:** Response values for different independent factors.

Run	Independent Factors		Average Removal of Antibiotics			
	Initial Concentration (mg/L)	Contact Time (day)	SMX (%)	SMX (mg L^−1^)	OFX (%)	OFX (mg L^−1^)
1	0.5	0.5	31.6	0.158	33.3	0.167
2	0.5	2.0	33.8	0.169	35.9	0.180
3	0.5	3.5	35.2	0.176	37.5	0.188
4	0.5	5.0	39.9	0.200	41.2	0.206
5	0.5	6.5	39.8	0.199	42.6	0.213
6	1.0	0.5	30.9	0.309	32.7	0.327
7	1.0	2.0	33.0	0.330	34.5	0.345
8	1.0	3.5	35.5	0.355	37.6	0.376
9	1.0	5.0	37.4	0.374	39.1	0.391
10	1.0	6.5	37.4	0.374	39.7	0.397
11	1.5	0.5	27.9	0.419	29.5	0.443
12	1.5	2.0	28.7	0.431	31.4	0.471
13	1.5	3.5	33.5	0.503	34.9	0.524
14	1.5	5.0	34.4	0.516	37.3	0.560
15	1.5	6.5	34.3	0.515	37.1	0.557
16	2.0	0.5	25.9	0.518	27.6	0.552
17	2.0	2.0	27.1	0.542	30.0	0.600
18	2.0	3.5	28.7	0.574	30.9	0.618
19	2.0	5.0	32.6	0.652	34.2	0.684
20	2.0	6.5	32.9	0.658	34.7	0.694
21	2.5	0.5	23.7	0.593	26.3	0.658
22	2.5	2.0	25.0	0.625	27.5	0.688
23	2.5	3.5	26.3	0.658	28.4	0.710
24	2.5	5.0	30.4	0.760	32.2	0.805
25	2.5	6.5	31.2	0.780	33.8	0.845
26	3.0	0.5	18.3	0.549	21.7	0.651
27	3.0	2.0	21.2	0.636	23.4	0.702
28	3.0	3.5	24.1	0.723	26.8	0.804
29	3.0	5.0	26.3	0.789	29.1	0.873
30	3.0	6.5	27.4	0.822	29.9	0.897

**Table 4:** Statistical analysis results for response parameters in RSM.

Resp.	Optimization with RSM					Final Equation (in Terms of Actual Mode) **
	R^2^ *	Adj. R^2^	Pred. R^2^	Adec. P.	SD	
SMX	0.982	0.975	0.960	42.52	0.85	32.50 − 1.06A + 0.08B − 0.90A^2^ − 0.08B^3^ ***
OFX	0.974	0.972	0.967	74.15	0.86	36.21 − 4.76A + 1.37B

* R^2^: Coefficient of determination; Adj. R^2^: Adjusted R^2^; Pred. R^2^: Prediction R^2^; Adec. P.: Adequate precision; SD: Standard deviation; and MSE: mean squared errors. ** Significant at “Prob > F” less than 0.05. *** A: Initial concentrations of antibiotics (mg L^−1^); B: contact time (d).

**Table 5:** Experiments for toxicological effects of antibiotics on microalgae.

Runs	Antibiotics Concentrations (mg L^−1^)	Time (day)	Runs	Antibiotics Concentrations (mg L^−1^)	Time (day)
1	0	1	25	0	5
2	10	1	26	10	5
3	20	1	27	20	5
4	30	1	28	30	5
5	40	1	29	40	5
6	50	1	30	50	5
7	0	2	31	0	6
8	10	2	32	10	6
9	20	2	33	20	6
10	30	2	34	30	6
11	40	2	35	40	6
12	50	2	36	50	6
13	0	3	37	0	7
14	10	3	38	10	7
15	20	3	39	20	7
16	30	3	40	30	7
17	40	3	41	40	7
18	50	3	42	50	7
19	0	4	43	0	8
20	10	4	44	10	8
21	20	4	45	20	8
22	30	4	46	30	8
23	40	4	47	40	8
24	50	4	48	50	8

## Data Availability

The data presented in this study may be available on request from the corresponding author.
